# Advances in Cryochemistry: Mechanisms, Reactions and Applications

**DOI:** 10.3390/molecules26030750

**Published:** 2021-02-01

**Authors:** Lu-Yan An, Zhen Dai, Bin Di, Li-Li Xu

**Affiliations:** 1 Jiangsu Key Laboratory of Drug Design and Optimization, China Pharmaceutical University, Nanjing 210009, China; 15651014368@163.com (L.-Y.A.); daizhen15@163.com (Z.D.); 2 Key Laboratory of Drug Quality Control and Pharmacovigilance, Ministry of Education, China Pharmaceutical University, Nanjing 210009, China

**Keywords:** freeze concentration, mechanism, accelerated reaction, frozen system

## Abstract

It is counterintuitive that chemical reactions can be accelerated by freezing, but this amazing phenomenon was discovered as early as the 1960s. In frozen systems, the increase in reaction rate is caused by various mechanisms and the freeze concentration effect is the main reason for the observed acceleration. Some accelerated reactions have great application value in the chemistry synthesis and environmental fields; at the same time, certain reactions accelerated at low temperature during the storage of food, medicine, and biological products should cause concern. The study of reactions accelerated by freezing will overturn common sense and provide a new strategy for researchers in the chemistry field. In this review, we mainly introduce various mechanisms for accelerating reactions induced by freezing and summarize a variety of accelerated cryochemical reactions and their applications.

## 1. Introduction

Early attention to frozen systems was mainly based on the practical problem of food storage in refrigerators. In recent years, scientists have paid more attention to cryosphere atmospheric chemistry and atmospheric-related freezing reactions on polar regions [1,2,3] and utilizing special low-temperature environments to develop more ways to deal with pollutants [4,5]. At the same time, with the development of biopharmaceuticals and reagents, a series of studies have been carried out on their stable and continuous storage as frozen systems [6,7]. However, in the field of chemistry, low temperatures are often not the preferred experimental conditions. In a frozen state, the rate of chemical reactions is decreased or even inhibited. The phenomenon that the reaction rate increases when the temperature decreases is counterintuitive, but some reactions in solution can be accelerated by freezing. This phenomenon has been described since the 1960s [8,9,10,11,12,13].

Although the mechanism of reaction acceleration in a frozen system is not fully comprehended, the freeze concentration effect is considered the main factor in many cases. Freeze-concentrated solution is seen as a reaction field [14,15]. The freeze concentration effect is the result of the conversion of water into relatively high purity ice crystals during freezing. Given ice is intolerant to impurities, and foreign atoms cannot enter ice lattice, all non-aqueous components escape to the freeze-concentrated solution and are concentrated in a reduced liquid phase. Crystallized solvents form in non-aqueous media in a frozen state. This leaves liquid pockets of highly concentrated solutes where reactions occur at a faster rate. Freeze concentration can produce a supersaturated solution in which the reactants can even be concentrated in 0.1% of the original liquid volume [16]. The acceleration caused by the concentration effect is greater than the inhibition caused by freezing, thus the final manifestation is that the chemical reaction is accelerated by freezing.

Since the 1960s, it has been known that freezing can accelerate certain chemical reactions. For example, the reaction rate of ethylene chlorohydrin and sodium hydroxide in was increased by 1000-fold in a frozen solution [17]. This phenomenon could be explained by the freeze concentration effect: at 5 °C, 0.001 M initial reactant concentration, 99.9% of the frozen solution was solid, and the reaction would proceed at 1000 times the initial rate in the remaining 0.1% of liquid. Besides, the study on the stability of penicillin amide bonds have found that β-lactam bonds were unusually cleaved in frozen systems [8]. Penicillin solutions containing imidazole lost antimicrobial activity when stored at −18 °C but not at 38 °C. The imidazole-catalyzed cleavage of the β-lactam bonds of penicillin only occurs in frozen systems at temperatures between −5 °C and −30 °C, not in supercooled systems. The concentration effect and the high proton mobility in ice promote the general base catalysis of β-lactam cleavage and rapid proton transfer in nucleophilic reactions. The structures of ice-like water containing “cavities” provide a rapid proton transport mechanism [8,10]. During this period, the discovery of many accelerated reactions in frozen systems aroused the interest of researchers. The traditional concept that reactions were inhibited at low temperature might thus be subverted and freezing might be considered as a new option for some reactions that were difficult to carry out at room or high temperature.

The accelerated cryoreactions can be classified into two categories: one is to break the traditional concept that chemical reactions can be hindered at low temperature. On the contrary, a known reaction is accelerated apparently as a result of freezing [18]. Usually, this type of reaction is explained by the freeze concentration effect. However, this kind of abnormally accelerated reaction should be taken seriously in the preservation of food, medicine and biological products. The other is new reactions that cannot proceed at room/high temperature, but can occur unexpectedly upon lowering of temperature. Freezing is a necessary condition for this kind of reaction to occur. This type of reaction is usually seen in peptide synthesis [19].

In conclusion, studying chemical reactions accelerated by freezing will overturn common sense, and provide novel knowledge for researchers in the chemistry field. This article mainly introduces the mechanism of accelerated cryoreactions and some accelerated cryoreaction examples to provide new exploration conditions for the reactions that are difficult to carry out at room or high temperature.

## 2. The Mechanism of Accelerating Reactions Induced by Freezing

Compared with reactions in solution, the rate of certain reactions may show interesting phenomena in frozen systems where solvent crystallization produces liquid-to-solid transitions [20,21,22,23]. What kinds of engine devices exist in the frozen system and how do they drive the acceleration of reactions? The mechanisms currently considered that have the main responsibility for accelerating reactions in frozen environments are as follows [18,24].

### 2.1. Freeze Concentration

**Figure 1:**
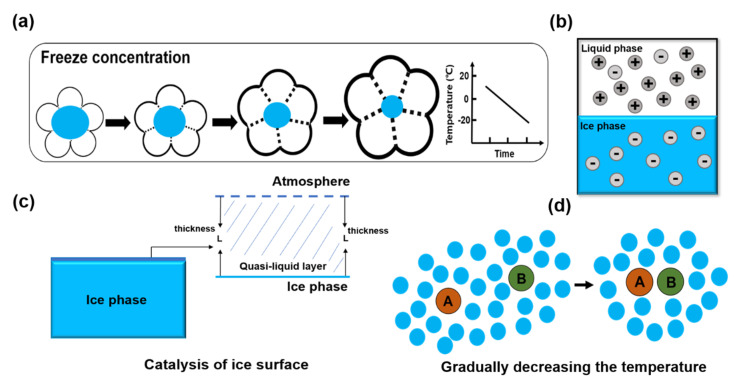
The mechanism of accelerating reaction in frozen system. (**a**) Freeze concentration effect. As the temperature decreases, the size of the liquid sites (colored in blue) decreases, so that the solutes are further concentrated [18,20]. (**b**) Freezing-potential effect. The unbalance of cations and anions incorporated into the liquid and ice phase results in the generation of electric potential. (**c**) Catalysis of ice surface effect. The surface of ice contains a liquid layer called quasi-liquid layer (QLL). Specific chemical reactions are greatly accelerated here. (**d**) The cage effect. As the temperature decreases, the average intermolecular distances among the solutes decreases.

**Scheme 1:**
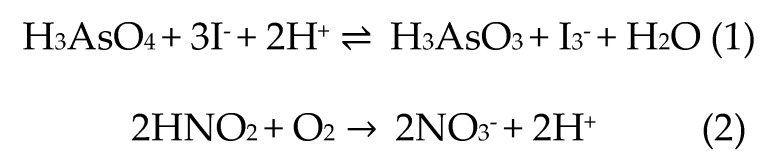
(1) The reaction of arsenic acid and iodide; (2) The reaction of the oxidation of nitrite by dissolved oxygen.

In frozen solutions, the solutes are expelled from the growing crystallized solvents and concentrate in the remaining liquid, as shown in Figure 1a. Freeze concentration is the main reason for the increasing reaction rates observed in frozen systems [25]. This effect was first described for the reaction of arsenic acid and iodide (Scheme 1) [12]. This reversible oxidation reaction could be accelerated in a frozen solution. The increase of reactants concentration caused by freezing increased the forward reaction rate. At the same time, the presence of iodide ions and protons reduced the reverse rate and the precipitation of iodine at low temperature further promoted the equilibrium shift to the right. The proposal of concentration effect provided a theoretical basis for future studies of accelerated reactions in frozen systems. Freeze concentration operates in the frozen state above the eutectic point where both liquid and solid phases co-exist. Cryochemical reactions below the eutectic point are also known. For example, the photochemical dimerization of thymine in ice was shown to be a solid-state reaction which occurred below the eutectic point [26,27]. Solutes and dissolved gases in the solution are push out by the solvent crystallization and are concentrated in the shrinking liquid phase. Freeze concentration causes not only an increase in the concentration of the solution, but also can alter the pH of solutions [28,29]. In the concentrated solution, protons and hydroxides accumulate in liquid brine under acidic and alkaline conditions, respectively. Therefore, acidic solutions become more acidic, while alkaline solutions become more alkaline as a result of freezing [30]. The concentration of solutes, dissolved gases and protons/hydroxides in liquid brine during freezing is called the “freezing concentration effect” [31]. It is currently believed that freeze concentration is the largest contributor to most accelerated reactions in frozen solution.

### 2.2. Freezing-Potential

Not all of solutes will be expelled from the growing ice phase. In contrast, some solutes are integrated into the ice phase. The difference and imbalance between anions and cations incorporated into the unfrozen solution and ice crystals generate an electric potential [32], as shown in Figure 1b. The freezing potential is produced by the transient charge imbalance in the unfrozen solution and the ice phase. Freezing potentials of ice has been reported to be +214 to −90 V [33]. This potential depends on the salt concentration and the type of ions involved in the solution. Highly electronegative anions can replace water molecules in the ice crystals, and molecules with similar structures to water are well incorporated into ice crystals. For example, a negative potential is generated when ice is in contact with the frozen solution containing F^−^. When a solution of an NH_4_^+^ salt is frozen, a positive freezing potential is obtained [34,35]. The freezing potential is mainly relaxed by the transfer of H^+^ or OH^−^ between the ice and the unfrozen solution. Freezing potential could influence reactions of environmental importance involving redox processes [36].

### 2.3. The Varies of pH Value Due to Neutralization of the Freezing Potential

The change of pH is an equilibrium phenomenon, not a transient process. The pH in the unfrozen solution is shifted because of the transfer of H^+^ or OH^−^ [34]. When an anion is better incorporated into the ice structure, the unfrozen solution is basified to maintain electroneutrality. On the contrary, the preferable incorporation of a cation into ice leads to the acidification of the unfrozen solution.

H^+^ or OH^−^ are transferred between ice crystals and the unfrozen solution to neutralize the freezing potential and then a hydrolysis reaction occurs. For example, the oxidative decomposition of gallic acid occurs in alkaline solutions, but hardly occurs in acidic solutions. Studies have found that even under neutral and acidic conditions, the addition of sodium chloride can promote the decomposition of gallic acid in frozen systems [37]. When a sodium chloride solution is frozen, chloride ions are more easily integrated into ice phase than sodium ions. The unfrozen solution becomes positively charged. Therefore, protons transfer from the unfrozen solution to the ice and the pH of an unfrozen gallic acid solution becomes alkaline and the decomposition of gallic acid can proceed. Also, pH changes by freezing involve the deposition of some buffer salts (a phenomenon particularly known for phosphate buffers), which induces a very large pH shift [7,38,39,40,41]. The freezing potential would affect the pH depression of the solution, and the oxidation of nitrite by dissolved oxygen to form nitrate was accelerated (Scheme 1).

### 2.4. Ice surface Catalysis 

The surface of ice is a chemically different phase and contains a liquid layer called quasi-liquid layer (QLL) [42,43,44], as shown in Figure 1c. The liquid layer has different characteristics from the bulk material because the molecules or atoms present in the layer only encounter with other molecules from one side [45]. Some atmospheric reactions are accelerated in QLL. Many experiments have confirmed that the photolysis of nitrate occurs in the QLL on the surface of ice crystals [46,47].

The ice grain boundary region contains a small fraction of liquid water and the liquid-like grain boundary layer may experience different reaction conditions (e.g., solute concentration, ionic strength, pH) from those of the aqueous solution. The solutes in frozen solutions are highly concentrated there. The enhanced photodissolution of iron oxides occurs in this confined boundary region compared with reactions proceeding in aqueous solution [48,49].

The ice wall or the water structure near the wall is also related to kinetic enhancement. Compared with liquid water molecules, the rigid intermolecular hydrogen bonds of the solid state should promote the dissociation of water molecules on the ice surface [50]. The dissociated ice wall may catalyze reactions. Besides, the reaction becomes faster as the volume of liquid inclusion decreases. For example, the complexation of crown ethers is greater in unfrozen solution at the same ionic strength and the same temperature [51]. This enhancement depends on the size of the liquid inclusion formed in the ice matrix. The hydrolysis of fluorescein diacetate also proceeds rapidly in an unfrozen solution [50]. Hydroxide ions can be transferred through the water structures by hopping. This should increase the catalytic ability of hydroxide ions for hydrolysis near the ice wall.

### 2.5. Chemical Equilibrium Shifts

If the number of reactants is different from the number of products, reversible reactions must undergo chemical equilibrium shifts due to a decrease in liquid volume caused by freezing. For chemical systems with reversible reactions, multiple equilibrium shifts will occur upon freezing [52]. Usually, the liquid volume decreases, and the concentration increases due to freezing. This is correct for: (i) the solutes that do not participate in the equilibrium reaction and (ii) the sum of all dissolved substances. This is not the case for the individual concentration of species participating in equilibrium reactions. Multiple equilibrium shifts lead to increases and decreases of concentration for solutes involved in equilibria during freezing. The influence of freezing on kinetics is closely related to concentration. So far, the influence of rapid equilibrium shift has not been considered in the theoretical development of reaction kinetics in frozen systems.

Newberg used a probe molecule in combination with in-situ spectroscopy and multiple equilibrium shift model to describe the effect of multiple equilibrium shifts on solute concentrations upon freezing [52]. Equilibria can also be studied using in situ measurements. For example, the alkaline-catalyzed hydrolysis of fluorescein diacetate is a pseudo-first order reaction at a constant alkali concentration [50,53]. At the same temperature and ionic strength, the reaction in a frozen state is faster than that in an unfrozen solution. The reaction rate cannot be evaluated by the freeze concentration effect. In situ fluorescence measurements are feasible for evaluating reaction rates and equilibria by the time change in the fluorescence.

### 2.6. The Cage Effect

The cage effect can be defined as the fraction of radical pairs that undergo reactions within a primary reaction cage [54]. The temperature has a strong impact on the cage effect, which increases as the temperature decreases. When liquid solutions start to solidify, organic molecules gather as self-organized hydrophobic clusters surrounded by water molecules. As the temperature decreases further, conformational motions and migrations are inhibited, and water molecules are further expelled from the clusters, shortening the average intermolecular distances among the solutes [55], as shown in Figure 1d. Lower temperatures and a more rigid environment place severe restrictions on diffusion. Poor molecular diffusion in the frozen solution alters reaction selectivity [50]. This effect determines, for example, the main product in the photodecarbonylation of dibenzyl ketones [54].

### 2.7. Convection Effect 

The freezing direction of the solution produces a concentration gradient.

### 2.8. Temperature Differences

Cooling a solution under laboratory conditions will cause temperature variations within the solution.

### 2.9. Conformational Changes

Low temperatures can reduce the flexibility of peptide backbones and promote the formation of the main conformation. 

In addition, the high proton mobility in ice, a favorable substrate-catalyst position constraint, diffusion of reactants and products, crystal defects, changes in dielectric behavior as well as conformational changes of enzymes all contribute to the increase of reaction rates under frozen conditions [24].

## 3. Kinetics of Reactions in Frozen Systems

**Figure 2:**
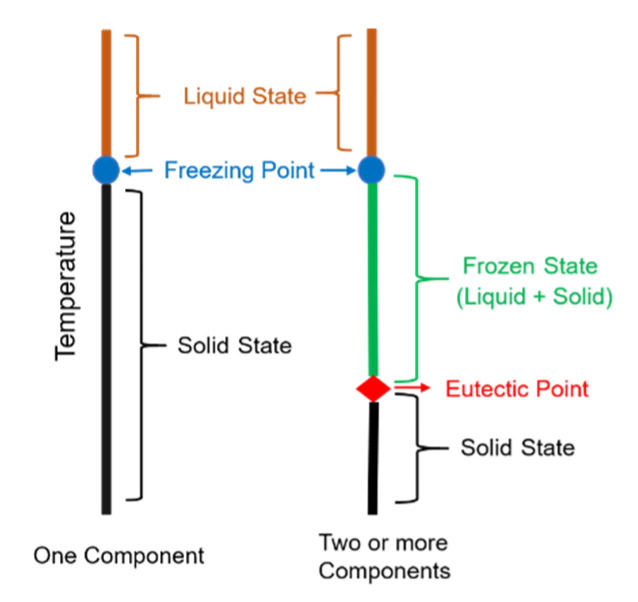
The transition of pure compound and solution or impure solid from liquid to solid or from solid to liquid over a range of temperature.

As show in Figure 2, a “frozen state” exists in the range of temperatures above the eutectic point and below the freezing point, where the solid phase is in equilibrium with the liquid phase [13]. Only below eutectic point, is the system completely solid. Frozen systems describe real systems where most of the cryochemical processes occur. It is worth noting that reactions in the solid, in the liquid, or in both phases must be considered at any temperature in the frozen state.

A freezing-induced high local concentration of substrates favors both the reaction thermodynamics and enhances the kinetics. Lowering the reaction temperature can weaken the entropy penalty and increase the equilibrium constant [56]. Pincock was the first to report a model for accelerated reactions in frozen systems [13]. Takenaka and Bandow proposed a modified version of the Pincock equation and the mechanism could only be applied to reactions driven by freeze-concentration effects.

The reactions accelerated by freezing are as follows [57]:

(1) Second- or higher-order reactions

For a first-order reaction, the rate equations of reactions in frozen systems are the same as those in solution. The reaction rate in an unfrozen solution follows the temperature dependence of the solution. Freezing cannot accelerate a first-order reaction. Pseudo first-order reactions are usually regarded as first-order reactions in solution because the concentration of one of the reactants is constant. In the calculation, the concentration of this reactant is combined with the rate coefficient.

(2) Low total initial concentration and low reactant concentration

When at low total concentrations, the reaction rates in unfrozen microphase solutions are much faster than those in solution. When the total concentrations are high, the reaction rates are not very fast compared with the reactions in the solution. At high total concentration, the ratio of the concentration of solutes in unfrozen microphase solution to the total concentration is low and the reaction rate is slow due to the low freeze-concentration effect for the reactant.

(3) Small activation energy of the reaction

The reaction rates in unfrozen microphase solutions at lower temperatures are much faster than those in the solutions at higher temperatures. The effects are more obvious for reactions with lower activation energy. The reaction rate coefficients have a maximum at the certain temperature. When the reaction has a lower activation energy, the temperature that exhibits the maximum reaction rate is lower or shows no maximum until the eutectic point.

The total concentration, the initial reactant concentration, eutectic point and the temperature exhibiting the maximum reaction rate are important factors to control the reaction rate in a frozen system.

In the above case, it is assumed that the reactants or products are determined after thawing. Additional effects maybe arise during the thawing process. If the reaction rate is independent of concentration, it cannot be explained by the freeze concentration effect. Reaction rates without thawing and equilibria can also be studied using in situ measurements [50,53]. However, there are some reactions that can take place driven by other mechanisms during freezing. For example, the variation of pH accelerates hydrolysis reactions [37], the ice wall catalysis phenomenon [50], the cage effect [55], and the solute migrates from the ice into the solution and reacts [54,58]. Here, we mainly explain the mechanism of the freezing-concentration effect.

The general kinetic model and respective kinetic equations developed by the team of Sergeev and Batyuk for chemical reactions in frozen multicomponent solutions give very good descriptions of real experimental results [59]. They summarize the kinetic features of chemical reaction at low temperature as follows: (1) The temperature dependence of reaction velocity is typical of frozen multicomponent systems. The transition of the system from a liquid phase to a frozen state is accompanied by an increase in the rate, that is, a negative temperature coefficient phenomenon is observed. (2) The most remarkable characteristic of the reactions in binary systems is a phase transition effect, which mainly occurs at low temperature without solvent. Most mixtures usually react when one of the components melts. In the absence of solvents, chemical reactions can occur at high rates at low temperature, and usually produce a single product in nearly the theoretical yield.

The kinetics of chemical reactions in frozen systems can be explained according to certain basic principles [59]: (1) The features of multicomponent systems are the inhomogeneity of structure and phase. (2) Phases with different molecular mobility coexist over a wide range of conditions. (3) Freezing is accompanied by changes in the physicochemical properties of the system. The physicochemical properties of different phases are obviously different, which cannot be simply extrapolated from the data of a homogeneous system. With the decrease of temperature, the multicomponent systems formed from the liquid state have both structural and phase heterogeneity. The systems consist of phases with different compositions, physicochemical properties and molecular mobility. They can be seen as regions composed of different components, so they have different physicochemical properties. Solutes are preferentially concentrated in some regions, and the solvent is concentrated in other regions. The occurrence of chemical reactions is caused by the phase inhomogeneity. This brings the reactants together to satisfy the important conditions for reactions to take place. Therefore, chemical reactions occur in certain regions of a frozen system.

Such a fraction of the moderately (non-deeply)-frozen multicomponent solutions, which is remained in a liquid state, was termed as the ‘unfrozen liquid microphase’ by Sergeev and Batyuk [59]. From a thermodynamic point of view, the formation of the liquid microphase in multicomponent frozen systems is due to the fact that inclusion of dissolved substances in the crystal lattice of solid solvent requires more energy than that required for the increase in the chemical potentials upon an increase in the concentrations of components in the liquid microphase [60].

Sergeev and Batyuk described in detail the kinetic equations of the reaction rates in the liquid microphase of frozen systems, the analytical expressions for the time variation of concentration of reactants or reaction products, and the extrema of the general temperature dependence of the reaction rate in the frozen solution [59]. At the same time, the influences of different factors on the reaction rate were reviewed, such as the concentration of solutions, inner additive, the concentrating factor, freeing point of the solution, the activation energy and the reaction order.

## 4. Various Reactions Accelerated in Frozen Systems

### 4.1. Hydrolysis Reactions

The spontaneous hydrolysis kinetics and catalytic hydrolysis kinetics of acetic anhydride and β-propiolactone in water and ice have been compared [61]. Compared with the same reaction in a liquid, the reaction rate of catalytic hydrolysis in the frozen system increased significantly. This was due to the fact that when the reaction mixture was frozen, the reactants could not be dispersed into the ice crystals but rather remain in the small liquid area between the ice crystals. This leads to the accumulation of catalytic substances and accelerates the reaction [9]. When an electrolyte was added to the reaction system, the liquid area increases and the reactants are diluted, resulting in a decrease in the reaction rate. Contrary to the catalytic hydrolysis reaction, the spontaneous reaction was suppressed in the frozen system, but when electrolytes such as potassium chloride were added, the increase in ionic strength greatly enhanced the spontaneous reaction rate in ice. The rate enhancement phenomenon could be explained by the increasing concentration of reactants in the liquid region. Here, the substrate and water were the only reactants in the spontaneous reaction. The addition of electrolyte would increase the size of the liquid area and increase the reaction rate.

The hydrolysis reaction of acid anhydrides and esters is similar, but the effects of frozen systems on catalytic hydrolysis and spontaneous hydrolysis are completely opposite. The main reason for this difference is whether water is used as a reactant. Freezing will greatly reduce the water content, thus decreases the rate of spontaneous reaction. In catalytic hydrolysis, water is not the reactant. Therefore, when the system is gradually frozen, the reactants are gradually concentrated in the reduced liquid so the reaction rate increases.

The hydrolysis rate of fluorescein diacetate (FDA) in the frozen state is several times faster than that in the solution of the same composition at the same temperature [50]. Unlike the above reaction, this is a pseudo first-order reaction and cannot be explained by the concentration effect. The enhancement in reaction rate is obviously related to the size of the space in which the liquid phase is confined during freezing. Therefore, the ice wall itself or the water structure formed near the wall should be responsible for this kinetic enhancement.

### 4.2. Oximation Reactions

**Figure 3:**
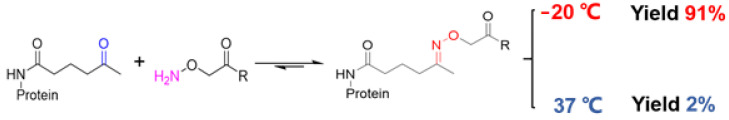
The reaction of oxime formation. The yield of oxime ligations is 2% at 37 °C, but the yield can reach up to 91% when the temperature is dropped to −20 °C.

The rate of oxime formation is very slow, so it is not commonly used for bio-conjugation. At neutral pH, the reaction of ketones or aldehydes with the aminooxy moiety forming oxime bonds is very slow, and it is often necessary to add an aniline catalyst or a derivative to accelerate the reaction [62,63,64]. However, it was lately found that under neutral pH, the oximation reaction can be accelerated when the reaction mixture is slowly frozen at −20 °C and the oximation reaction rate can increase by two orders of magnitude, which causes a more pronounced rate increase than the aniline catalyst [65], as shown in Figure 3. Possible mechanisms for this phenomenon are: (1) a concentration effect caused by freezing; (2) ice crystal growth may have a catalytic effect; (3) an increase of salt concentration results in changes in pH and ionic strength; (4) chemical thermodynamics require that the reaction shift strongly to the right because the volume of water decreases.

This discovery is a big breakthrough for the oximation reaction. Nevertheless, freezing catalytic reactions has certain limitations. For example, it is unsuitable for proteins that are unstable and aggregate during freezing. Compared with traditional catalysts like aniline and its derivatives, a frozen system does not accelerate the reverse hydrolysis, does not require complex purification, shortens the reaction time and is more environmentally friendly.

### 4.3. Peptide Synthesis

#### 4.3.1. Enzyme-Catalyzed Synthesis of Peptides

**Figure 4:**
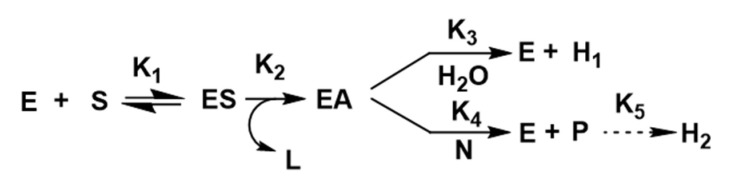
Protease-catalyzed kinetically controlled peptide synthesis. E, enzyme; S, acyl donor ester; ES, Michaelis complex; EA, acyl enzyme: L, acyl donor leaving group; H_1_, hydrolysis product; P, peptide product; N, amino component; H_2_, peptide hydrolysate.

At present, the methods of synthesizing peptides mainly are enzymatic and chemical methods. Compared with chemical methods, enzymatic synthesis has the advantages of mild reaction conditions, high regioselectivity and low toxicity. Enzyme-mediated synthesis of peptides is gradually becoming a more sustainable alternative than chemical methods [66,67]. In the late 1970s, the conversion of porcine insulin to human insulin aroused great interest in this field [68]. To improve the yield of enzyme-catalyzed peptide syntheses different chemical reaction conditions were tried. In the 1990s, Hänsler proposed a frozen water reaction system. As shown in Figure 4, the efficiency of peptide bond formation is affected by the competitive hydrolysis of the acyl enzyme [16]. The other peptide yield-limiting factor is the hydrolysis of peptide products. In a frozen system, the decrease in liquid volume can produce a concentration effect. At the same time, the reduction of water concentration inhibits the hydrolysis of acyl enzymes and peptide products, so frozen systems can increase the yield of peptide [16].

**Figure 5:**
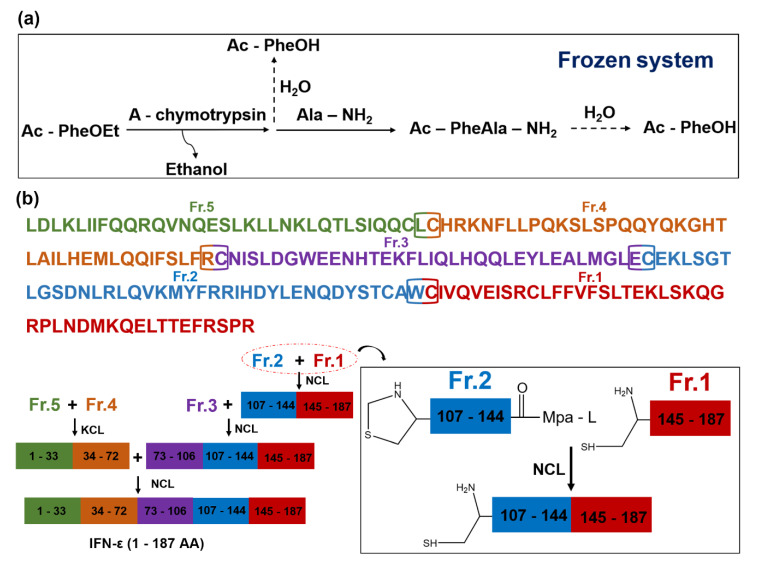
Peptides synthesis at low temperature. (**a**) Ammonolysis reaction of N-acetyl-L-phenylalanine ethyl ester (Ac-PheOEt) and L-alaninamide (Ala-NH2). (**b**) The strategy for the convergent synthesis of the hIFN-ε protein (187 AA) based on native chemical ligation.

Jönsson studied the synthesis of dipeptides, the ammonolysis reaction of N-acetyl-L-phenylalanine ethyl ester (Ac-PheOEt) and L-alaninamide (Ala-NH_2_) [69], as shown in Figure 5a. Four different water-miscible solvents were used. The main difference of the four solvents at low temperatures was that acetonitrile and tetrahydrofuran (THF) could crystallize with the formation of the respective solids, while acetone and diglyme did not. In the four solvents, the peptide yield increased with the decrease of temperature. The yield changes in acetonitrile and THF were more obvious than the changes in acetone and diglyme. Studies found that the substrate Ac-PheOEt released ethanol to acylate the enzyme and formed an acyl-enzyme complex. The nucleophile (Ala-NH_2_) competed with water for the deacylation, and formed two possible products, the dipeptide and acid. Nucleophiles had a stronger competitive effect than water at low temperatures. Therefore, with a fixed concentration of nucleophile, higher peptide yields could be obtained at lower temperatures. However, after the peptide was formed, the enzyme could continue to hydrolyze the products. Therefore, studying the esterase and amidase activities of α-chymotrypsin at low temperature conditions had a certain significance to increase peptide yield. Studies have shown that at low temperature, the hydrolysis of esters was more favorable, and the hydrolysis of dipeptides was significantly inhibited, so low temperatures promoted the synthesis of dipeptides by two methods: first, nucleophiles had a stronger ability to compete with water to deacylate acyl-enzyme complexes; Second, ester substrates were more capable of competing for free enzymes than peptide substrates.

Compared with room temperature reactions, enzymatic peptide synthesis in frozen systems is more conducive to the synthesis of target peptides and greatly reduces the formation of byproducts. Taking advantage of this, the synthesis of poly-L-cystine catalyzed by α-chymotrypsin in frozen aqueous solution has been achieved [70]. Freeze-concentration [71], catalysis on the ice surface [50,51], equilibrium shift [52], the variations of pH value, the interaction between enzymes and ice, changes in the physical and chemical properties of enzymes, favorable substrate and catalyst orientation and reduced water activity [16] in frozen solutions also have non-ignorable impacts in the synthesis of target peptides. These all are possible reasons which might increase the peptide yield [72].

#### 4.3.2. Chemical Synthesis of Peptides

The chemical synthesis of peptides is still the preferred method in the laboratory, especially solid phase peptide synthesis (SPPS) [73,74,75,76]. In order to synthesize peptides with different lengths and complex designs, various experimental conditions have been optimized and tried, such as the use of different solvents [77], diverse coupling reagents [78], ultrasound assistance [79], and low temperatures [80]. Low temperatures not only increase the yield of enzymatic peptide synthesis, but also have a very significant effect on the chemical synthesis of peptides. In 1997, Vajda’s laboratory found that the DCC-mediated peptide coupling reaction could occur successfully in frozen organic solutions and the formation of by-products could be significantly inhibited at the same time. The coupling of peptides in frozen organic solutions opened up a new way for the chemical synthesis of peptides [80].

In the process of chemical total synthesis of human interferon-ε, our laboratory recently discovered that low temperature could induce peptide ligation [19]. Human interferon-ε (hIFN-ε) is composed of 187 amino acids, which can be prepared by synthesizing five peptide fragments of similar length and ligating them four times, as shown in Figure 5b. Among them, when the Fr1 (107–144) and Fr2 (145–187) fragments were joined, no ligation product was detected after a period of reaction under typical NCL conditions [81]. Subsequently, the experimental conditions were optimized, including extending the reaction time, changing the pH and increasing the temperature. However, all these new attempts still failed to produce the target peptide. To verify that the specific peptide sequence near the junction makes it an unusable ligation site, two peptides with a length of about 10 amide acids located on both sides of the connection site were synthesized: one was the N-terminal cysteine-containing segment of Fr.1 [CIVQVEISRC(Acm)L and the other was the C-terminal thioester of Fr.2 [ENQDYSTC (Acm) AW-Mpa-L. These two short clips could easily achieve almost complete connection within 8 h under typical NCL conditions, which refuted the above hypothesis. Therefore, the length of the two peptide fragments was the main reason for the connection failure. For this peptide, the length of the sequence affected the secondary structure and steric hindrance. The junction between Fr.1 and Fr.2 was located just near the turning point between the helix structure (for the peptide structure readers can refer to [19]), so finding the dominant conformation might help the ligation of Fr.1 and Fr.2. Low temperature can reduce the flexibility of the peptide backbone and promote the formation of the main conformation [82]. Therefore, when the reaction temperature was dropped to −20 °C, the reaction product was successfully detected overnight [19]. In the process of overcoming the unreasonable condensation failure between Fr.1 and Fr.2 peptides, it was shown that freezing promoted the formation of the dominant conformation and further induced the connection of peptides. The formation of the main conformation is a supplement to the low-temperature chemical catalytic mechanism and is expected to contribute to the overall synthesis of biological macromolecules. In addition to peptides, studies have shown that freezing can also inhibit the degradation of RNA and makes the synthesis rate of RNA surpass the degradation rate [83,84,85].

The essence of peptide synthesis is the dehydration condensation reaction between the amino and carboxyl groups of amino acids. Therefore, in the process of freeze-driven peptide synthesis, the influence of equilibrium shifts on the reactions must be considered. First, under frozen conditions, the content of water decreases due to the icing effect. The dehydration condensation of amino and carboxyl groups further shifts to the right to promote peptide synthesis. Secondly, the hydrolysis reaction competing with the ammonolysis is suppressed. Third, after the peptide is formed, it may be hydrolyzed. The decrease of water inhibits the hydrolysis of the formed peptide in frozen systems. Therefore, in frozen systems, the equilibrium shift is a non-negligible factor for driving peptide synthesis.

From this point of view, whether enzymatic or chemical synthesis of peptides is considered, freezing can increase the yield of peptides through special mechanisms. In a low temperature environment, enzymes are not prone to inactivation and peptides can also exist stably. Therefore, low temperatures are an ideal choice for peptide synthesis. In the connection of peptide chains, we have to consider whether their conformation is conducive to linking. Therefore, the study of peptide conformations at low temperature is of great significance to the synthesis of peptides.

#### 4.3.3. Conformational Changes of Peptides

In various secondary structures of peptides, the α-helix is the most common conformation [86,87,88]. Approximately 40% of peptides in solution exist as α-helical structures [89]. As we know, the conformation is a critical factor affecting the molecular recognition and interaction between biomolecules [90]. The conformation of peptides is sensitive to temperature, and peptides can exist in their stable conformation at low temperatures. Many studies have found that peptides mainly exist in the helix form at low temperature and the helix content would increase with the decrease of temperature [91,92,93,94,95,96,97,98].

The synthetic peptide (PTN112–136), the last 25 amino acids in the C-terminal region of the pleiotropic growth factor (PTN), has a highly flexible peptide backbone in solution at room temperature. However, at lower temperatures, the flexibility of the skeleton was significantly reduced, and a fairly clear structure was adopted [99]. Lower temperatures can reduce the flexibility of the peptide skeleton and facilitate the formation of its main conformation. The secondary structure of peptides determines the type of surface approachable amino acid between two sequences. Therefore, the spatial orientation of the ligation site and the chemical reactivity of the given peptides are decided by the secondary structure [19].

### 4.4. Cryopolymerization Reactions

In addition to the reactions involving low-molecular reagents, there are numerous processes with the participation of high-molecular compounds or resulting to their formation via cryopolymerization reactions. Cryopolymerization is a powerful tool to produce inter-connected macroporous materials at a rapid rate and in high capacity. In the process of cryopolymerization, the frozen polymerization medium is used as the porogen, and the unfrozen liquid microphase is the polymerization site for constructing the obtained cryogel skeleton. The volume fraction of unfrozen liquid microphase is about 0.1, producing a freeze concentration effect [100]. After thawing, the melting of the frozen medium leaves holes among the polymer pore wall frames. Generally, cryopolymerization is carried out in water, and the temperature should be −15 °C or lower. Haleem et al. prepared macroporous oil adsorbents with high absorption capacity and high temperature tolerance through cryopolymerization [101]. Macroporous hydrophobic P(LA-co-EGDMA) cryogels was prepared through cryopolymerization of lauryl acrylate and ethylene glycol methacrylate as the crosslinking agent below the freezing point of the mixture.

Polymer cryogels are formed by freezing of low or high molar mass precursors, crosslinking, and subsequent thawing [102,103]. Polymeric cryogels are generated when the gel-formation occurs in the frozen system, and the 3D polymeric network formed becomes swollen in the defrosted solvent during thawing [104]. Chemical crosslinking through redox systems is one of the most commonly used approaches for synthesis of polymer cryogels. Cryogels possess macroporous systems, because the polycrystals of frozen solvent act as the porogens, and such macropores are usually interconnected [60]. During the freezing process, a liquid–solid phase transition takes place and the initial solvent crystallizes. Therefore, the formed polycrystals or ice fulfill the porogen function and the initial precursors are concentrated in the unfrozen liquid microphase. This cryoconcentrating effect is considered to be one of the main driving forces for the cryotropic gel-formation. That is why the used solvent must be crystallizable rather than vitrified [104].

Macroporosity and interconnected pore systems are the characteristic features of the cryogenically-structured polymeric matrices. The interconnections of macropores are due to the contacts between the growing solvent polycrystals during freezing. During the thawing stage, these contacts turn into “interlinks” between the macropores. Lozinsky has discussed in detail numerous factors that affect the size (cross-section) and shape of the large pores [60,104]. One of the main effects of the cryotropic gel-formation processes is that the initial concentration of the precursors is obviously low, and the respective precursor in the unfrozen liquid microphase has cryoconcentration effect. Another effect is closely related to the cryoconcentration phenomena. The acceleration of the cryotropic gel-formation occurs over a certain range of negative temperatures.

The most known examples are the polyvinyl alcohol-based cryogels: the concentrated aqueous solutions of this polymer experience a sol-to-gel transition at low positive temperatures [60,104,105,106]. After several days’ incubation, low-melting (30–35 °C) and very weak gels were formed. A single freeze–thaw cycle allowed elastic cryogels to be obtained, and their fusion temperatures were markedly higher (60–80 °C) [105]. The main reason for this acceleration effect is the cryoconcentration of the gel-precursors in the unfrozen liquid microphase. In addition, in the process of redox-initiated copolymerization of acrylamide with N,N’-methylene-bisacrylamide (molar ratio of 30:1) in 3% aqueous solution at +20 °C, the gel-point (about 10% of gel-fraction yield) was reached after about 90 min, while in the frozen system at −20 °C, it took only 15 min, that is, about a six-fold acceleration was observed [104,107]. The bell-like temperature dependence of the freezing process is related to the linear cryopolymerization of the unsaturated monomers [104]. The reaction rate, the yield of the target polymer and the molecular weight of the obtained polymer are extremely dependent. The chain length of the polymers formed in the moderately-frozen system is usually significantly higher than that of the polymers prepared from the same initial reaction solution but at the temperature above the freezing point [104].

In addition, the studies on the cryopolymerization of aniline and pyrrole in frozen aqueous systems is also of great importance as the processes resulting in the synthesis of conducting polymers. Multifunctional thermal responsive conductive hybrid cryogels composed of poly(N-isopropylacrylamide) cryogel and conducting polyaniline or polypyrrole nanoparticles have many advantages, such as rapid shape memory properties, photothermal properties and pressure dependent conductivity [108,109].

Besides, rapid and controlled ring-opening polymerization of 1,2-dithiolanes under cryoconditions can be initiated by proteins bearing a reactive cysteine [56]. Decreasing the reaction temperature would weaken the entropy penalty and boost the equilibrium constant (Keq) based on the Van’t Hoff plot. Besides, frozen-induced high local concentration of substrates is clearly observed. The frozen reactions provide a plausible product band with high initiation efficiency compared with those occur at room temperature.

Preparation of materials based on proteins can be performed using a cryogelation technique, where the reaction proceeds in the frozen state and produces a supermacroporous material with the help of ice crystal formation [110].

### 4.5. Other Reactions and Applications

Since the cold areas of the world are natural frozen systems, more and more researchers are devoting themselves to studying cryoreactions for the degradation of pollutants in cold regions. Making full use of the environmental conditions in cold areas can not only reduce the difficulty of controlling reaction conditions, but also save money and energy. These accelerated reactions may become important weapons for protecting ecological environment in cold areas in the future.

**Scheme 2:**
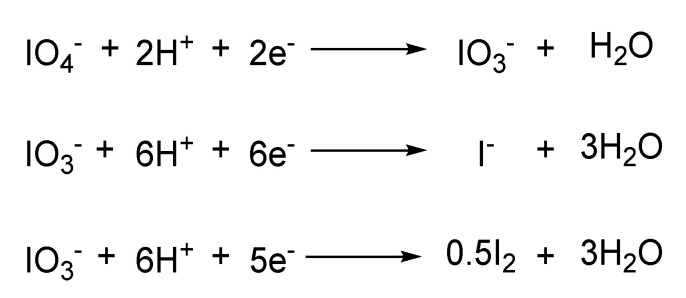
Electrons transfer from organic pollutants to IO_4_^−^ and IO_3_^−^.

Recent studies have shown that in a −20 °C environment, IO_4_^−^ can degrade furfural (FFA) and other organic pollutants (tryptophan, 4-chlorophenol, bisphenol A and phenol) [31,111]. These organic pollutants (i.e., pharmaceutical and phenolic compounds) are frequently used to evaluate a new water treatment system [111,112]. The degradation of these organic substances cannot be carried out in water at 25 °C. This unique behavior is caused by the freezing concentration effect, which concentrates the IO_4_^−^, organic pollutants and protons in the liquid brine between the ice crystals and provides a favorable position for the degradation process of proton coupling. Electrons transfer from organic pollutants to IO_4_^−^ during freezing, which leads to the degradation of organic pollutants and produces IO_3_^−^, as shown in Scheme 2, top reaction [31]. Electrons are transferred from organic pollutants to IO_3_^−^. IO_3_^−^ is further converted into I^−^ and I_2_, as shown in Scheme 2, middle and bottom reactions [31]. Therefore, not only IO_4_^−^ can be activated under freezing conditions, but also IO_3_^−^ produced from IO_4_^−^ reduction can also be activated [31]. Therefore, compared with other methods of activating the same dose IO_4_^−^, freezing can degrade more organic pollutants.

Sulfonamide drugs are widely used worldwide. After oral administration, approximately 45–70% of the intake is excreted within 24 h [113]. Therefore, a large amount of sulfonamides drug are discharged into the environment through sewage treatment systems or animal feces. Sulfonamides have received special attention due to their high concentrations and related risks in the environment. Sulfamethoxazole can undergo hydroxylation and non-biological transformation of nitrobenzene in the presence of nitrate [114]. Recent studies have shown that nitrite-driven conversion of sulfamethoxazole (SMX) may be negligible at ambient temperature, but non-biological nitrification, hydroxylation and oxidation reactions of SMX could be significantly accelerated in the ice phase and at the same time, the oxidation of nitrite to nitrate at low temperature can also be accelerated. This phenomenon is the result of the freezing concentration effect and freezing potential effect. When the dilute salt solution is frozen, extremely high concentrations of SMX and nitrite are produced (the freeze concentration effect). Secondly, a strong potential difference can be established between the ice crystal boundary and the ice phase. When the pH is lower than 9.5, the uneven distribution of various ions (such as Na^+^, PO_4_^3−^, NO^2−^) between the ice and aqueous phase leads to localized pH changes. At low pH, nitrous acid exists in the form of free nitrous acid, which will form reactive nitrogen and oxygen species, including nitric oxide (NO), OH radicals and hydrogen peroxide (H_2_O_2_)-like compounds. They may play an important role in SMX transformation [115].

Freezing promotes the reactions, which is rewarding for the treatment of pollutants in cold regions in a more energy-efficient and greener way. The discovery of these reactions is a big breakthrough in maintaining the ecological environment of cold region. First, it does not require additional conditions to create low-temperature environment, which is very economical; second, the efficiency of these accelerated reactions is very high, which greatly saves raw materials and treatment time; at the same time, these reactions will not produce new pollutants, and their treatments are more thorough. Therefore, these valuable low-temperature accelerated reactions not only are new discoveries in the field of chemistry, but also have important significance in the field of environment.

### 4.6. Undesirable Reactions and Changes

Generally, chemical reactions become slow in frozen systems. Therefore, we store food in the refrigerator to slow down the deterioration process, but recent studies have found that the reaction of dimethylamine and nitrite to produce N-nitrosodimethylamine (NDMA), which is a carcinogen, could be accelerated when it occurs in frozen concentrated solutions. Sodium nitrite is often added to processed meat and many amines are used as food color additives. If nitrosation is carried out counterintuitively by freezing, dangerous amounts of nitrosamines may be produced. Therefore, studying the N-nitrosation reaction in frozen systems is very important for the cryopreservation of food [29].

For most protein drugs, preventing and reducing protein aggregation is the key to maintain the stability of protein drugs. Protein drugs often need to be stored for a long time under frozen conditions, and the mechanism of protein inactivation in this condition is mainly aggregation [116]. Therefore, how to stabilize stored protein drugs or biological reagents at low temperatures has attracted the attention of researchers [117]. When an aqueous solution containing monosodium and disodium phosphate is frozen, the selective crystallization of disodium hydrogen phosphate will cause the pH of the freeze concentrate liquid to decrease, which can cause proteins to degrade [41]. Stabilizing the pH of the buffer solution by using “non-crystalline” buffer salts to prevent protein aggregation is an ideal method. In addition to protein drugs, the stability of amorphous drugs in frozen aqueous solutions has also received attention [118,119].

In daily life, we often use frozen systems to store perishable products. Therefore, studying the undesirable accelerated reaction and the changes of food, protein products and drugs at low temperatures are very important for extending the storage period and maintaining the safety of food, biological reagents and drugs.

## 5. Conclusions

In frozen systems, solids and solutions coexist under thermodynamic equilibrium in a wide temperature range. As a special reaction condition, freezing speeds up some chemical reactions through a special mechanism based on freeze concentration. In recent years, the equilibrium shifts in frozen solutions have attracted interest, which have significant implication to our understanding of the “freezing concentration” phenomenon [52]. Surprisingly, freezing is helpful for the total synthesis of biomacromolecules. Freezing reaction media is an effective way to reduce side reactions and increase peptide yields. This intriguing and rare phenomenon subverts the traditional concept and provides us with new knowledge of cryochemistry. Grafting-from (GF) is an important yet underdeveloped strategy for protein−polymer conjugates [120,121]. Simple and efficient GF polymerization strategies that can grow biodegradable polymers from native proteins are highly desirable. Cryopolymerization of 1,2-dithiolanes achieves the facile and reversible grafting-from [56]. The unique characteristics of frozen solutions can be used to develop novel reaction systems. These successful applications should be the motivation for more research on frozen systems.

Cryochemistry is closely related to many fields. Cold regions have natural frozen systems, and the discovery of more accelerated reactions is advantageous for environmental protection and atmospheric research. The impure ice acts as a cryoreactor, governing the circulation of chemical species of environmental importance [53]. Freezing can also be a unique method of base-catalyzed reactions without adding any alkaline reagents under neutral or acidic conditions. Freezing occurs on a large percentage of the Earth’s surface, and NaCl also exists everywhere. NaCl would have a great influence on alkali-decomposition of humic acids in the frozen environment [37].

Besides, the frozen system is used as a storage environment for food, drugs and biological reagents. The changes in physical properties or the counterintuitive accelerated reactions caused by freezing can affect the quality and safety of stored items. Therefore, future research on frozen systems may focus on discovering more favorable accelerated reactions and new mechanisms for promoting reactions. It is also necessary to optimize the storage conditions of food, drugs and biological products in frozen systems to ensure their stable and long-term storage. This review summarizes the mechanisms of accelerating reactions and different kinds of accelerated chemical reactions in frozen system, which provides ideas for in-depth research on cryochemistry in the future.

## Data Availability

The data presented in this study are available in this article.
